# Segmentation of Rhythmic Units in Word Speech by Japanese Infants and Toddlers

**DOI:** 10.3389/fpsyg.2021.626662

**Published:** 2021-04-09

**Authors:** Yeonju Cheong, Izumi Uehara

**Affiliations:** ^1^Department of Psychology, Ochanomizu University, Tokyo, Japan; ^2^Institute for Education and Human Development, Ochanomizu University, Tokyo, Japan

**Keywords:** word segmentation, intermodal matching procedure, syllable, mora, Japanese infants

## Abstract

When infants and toddlers are confronted with sequences of sounds, they are required to segment the sounds into meaningful units to achieve sufficient understanding. Rhythm has been regarded as a crucial cue for segmentation of speech sounds. Although previous intermodal methods indicated that infants and toddlers could detect differences in speech sounds based on stress-timed and syllable-timed units, these methods could not clearly indicate how infants and toddlers perform sound segmentation. Thus, the present study examined whether Japanese infants and toddlers could segment word speech sounds comprising basic morae (i.e., rhythm units similar to syllables), on the basis of concurrent basic mora units within syllable units, using the new intermodal matching procedure. The results indicated that, regardless of their ages and linguistic abilities, Japanese infants and toddlers aged 6–25 months tended to segment Japanese words comprising basic morae sounds on the basis of concurrent basic mora units within syllable units. This implies that infants' and toddlers' use of syllable units for segmentation of speech sounds at an early age could be evident among many infants and toddlers learning various languages. Although this finding should be interpreted carefully, the present study demonstrated the utility of the new intermodal matching procedure for examining segmentation of speech sounds and word sounds by infants and toddlers, on the basis of specific rhythm units.

## Introduction

When infants and toddlers are exposed to vocal speech, they are confronted with sequences of sounds and are required to segment the sounds into meaningful units to achieve sufficient understanding. Segmentation of speech might constitute a fundamental cognitive skill for an infant or a toddler to develop linguistic abilities. Previous research has focused on how infants and toddlers recognize the boundary of a sound within speech and when they begin to segment meaningful word sounds from the flow of speech. Segmenting speech sounds is presumably enabled by the perception of several linguistic cues, such as rhythmic cues (e.g., Echols et al., 1997; Johnson and Jusczyk, 2001), allophonic cues (e.g., Jusczyk et al., 1999a), phonotactic cues (e.g., Mattys and Jusczyk, 2001), and transitional probabilities between syllables (e.g., Saffran et al., 1996).

Infants begin to use these cues to segment speech sounds until ~1 year of age. Notably, rhythmic cues have been identified as a crucial component of speech sounds (Echols et al., 1997; Johnson and Jusczyk, 2001; Goyet et al., 2013). There are reportedly two main types of rhythmic cues (Dauer, 1983; Ramus et al., 1999; Arvaniti, 2009): stressed-timed and syllable-timed rhythms. Although mora-timed rhythm can be distinguished from these two rhythm categories (Abercrombie, 1967; Hoequist, 1983), this rhythm is also considered a type of syllable-timed rhythm (Otake et al., 1993; Arvaniti, 2009; Mazuka, 2009) according to indexes such as pairwise comparisons of successive vocalic and intervocalic intervals introduced by Grabe and Low (2002).

A mora is a segmental minimum unit of rhythm (Dan et al., 2013; Ogino et al., 2017) and is represented in the rhythmic structure of the Japanese language. A mora usually consists of a CV (consonant, vowel) or V syllable, which are called “basic morae.” More than 70% of Japanese sound units are basic morae, and can be segmented in a manner identical to that of syllables. However, a small number of morae, termed “special morae,” include special patterns of syllables “such as a nasal coda (CVN or VN), a geminate stop consonant (CVQ or VQ), a long vowel (CV: or V:), or a contracted sound (CjV)” (p. 113, right column, lines 6–8 from Ogino et al., 2017). Special morae are segmented in a manner that differs from that of syllables. For example, kitte (meaning stamp) includes three morae (ki, t, and te: “t” is a special mora unit, while “ki” and “te” are basic mora units) but two syllables: “t” is not counted as a syllable unit, while “kit” and “te” are considered constituent syllables.

Here, we briefly review past studies of speech sound segmentation in infants and toddlers for each of the three rhythm categories. Infants and toddlers whose native language belongs to the stress-timed category (e.g., English, German, and Dutch) use trochaic (strong-weak word stress pattern) or iambic rhythms (weak-strong word stress pattern) to segment speech sounds. Infants learning stress-timed languages begin to use or prefer trochaic units earlier than iambic units (Jusczyk et al., 1993). For example, English-learning 7.5-month-old infants segmented more often with a trochaic rhythm, rather than an iambic rhythm (Jusczyk et al., 1999b). Six-month-old German-learning infants also demonstrated this tendency (Höhle et al., 2009). Both Dutch- and English-learning 9-month-old infants could segment trochaic Dutch words in passages (Houston et al., 2000). The earliest age at which infants segment using an iambic rhythm as often as a trochaic rhythm is 10.5 months of age (Jusczyk et al., 1999b).

Infants and toddlers whose native language belongs to the syllable-timed category (e.g., French, Spanish, and Catalan) use syllable units to segment speech sounds. Nazzi et al. (2006) indicated that French-learning 12- and 16-month-old infants and toddlers (but not 8-month-old infants) could detect disyllabic words, which had been presented in isolation 15 times or embedded in several passages during the familiarization phase, although methods of detection or segmentation tended to differ between 12- and 16-month-old infants and toddlers. When the daily language environment involved Catalan only, Spanish only, or both languages, 6- and 8-month-old infants could detect monosyllabic words in passages (Bosch et al., 2013). Furthermore, Nishibayashi et al. (2015) indicated that, in a test phase, 6-month-old infants could identify monosyllabic words and syllables embedded in disyllabic words that had been presented during a preceding familiarization phase.

There is minimal evidence regarding how infants and toddlers perceive and discriminate mora units in a continuous speech stream. French-learning infants could discriminate English and Japanese sentences within 5 days of birth, despite low-pass filtering of the stimuli (Nazzi et al., 1998). Yoshida et al. (2010) examined the non-linguistic trochaic and iambic tones for Japanese- and English-learning infants. Although neither had any preferences for either tone type at 5–6 months of age, English-learning 7–8-month-old infants could differentiate the two tones on the basis of preferences for trochaic tones, whereas Japanese-learning 8-month-old infants did not show any preference for trochees or iambs. Nevertheless, there has been no direct examination involving whether or how Japanese-learning infants and toddlers segment speech sounds using rhythm cues in mora-timed language. As noted above, a mora-timed rhythm is also a presumptive type of syllable-timed rhythm (Otake et al., 1993; Arvaniti, 2009; Mazuka, 2009). Indeed, 2-month-old English-learning infants could differentiate passages in English from passages in Japanese, although they could not differentiate passages in French from passages in Japanese (Christophe and Morton, 1998).

While infants' and toddlers' responses toward specific rhythm types have been examined, several studies have proposed that infants and toddlers have broad speech perception in terms of syllables (e.g., Bertoncini et al., 1988; Jusczyk et al., 1995; Räsänen et al., 2018). Many languages are composed of a CVsound, which is a basic syllable unit. Nearly half of the sound forms in English are the CV, V, or VC form. Approximately 80% of the sound forms in French are CV, V, or VC, while more than 70% of the sound forms, so-called “basic morae” (as mentioned above) in Japanese are CV or V (Greenberg, 1999; Dankovičová and Dellwo, 2007). The syllable unit is considered a universal unit for examination of language structure (Mehler et al., 1981). Because French and Japanese, especially, have higher amounts of similarity in basic syllable structure and high degrees of phoneme regularity, French words could be relatively easily adapted to Japanese basic morae (Shinohara, 1996). Other previous studies have examined language learning by infants with respect to syllable units (e.g., Mehler, 1981; Bijeljac-Babic et al., 1993).

The prosodic bootstrapping hypothesis holds that prelinguistic infants acquire prosodic aspects of language including stress, rhythm, and intonation, which they use to identify speech sound boundaries and grammatical features (e.g., Gleitman and Wanner, 1982; Jusczyk, 1997; Soderstrom et al., 2003; Bernard and Gervain, 2012; Wellmann et al., 2012). Whereas some studies have indicated that infants younger than 12 months may perceive language-specific grammatical features such as word order (e.g., Japanese and Italian 8-year-olds in Gervain et al., 2008), some other studies have indicated that infants may develop skills regarding general phonological perception of speech sounds at an earlier age (e.g., Jusczyk, 1997; Soderstrom et al., 2003). For instance, a small number of Japanese studies have suggested that rhythm segmentation skills concerning Japanese “special morae” sounds may develop long after the infantile period, at around 4 years of age (Takahashi, 1997, 2001). Considering these findings, we assumed that more general basic segmentation skills that are widely shared, regardless of language and linguistic skills, and can be framed in terms of syllables, would develop earlier than language-specific perceptual and segmenting skills (which might be more closely related to the acquisition of specific first words), and that they would remain after acquiring the first words. In the present study, we focused on the basic segmentation skills that are expected to develop earlier. However, general segmentation skills have not been directly examined or empirically confirmed using previous methodologies.

Past methods frequently used the headturn preference procedure (e.g., Houston et al., 2000; Nazzi et al., 2006; Höhle et al., 2009; Bosch et al., 2013; Nishibayashi et al., 2015). These methods could not directly demonstrate the method of speech sound segmentation by infants and toddlers. The typical headturn preference procedure is as follows. In the booth where the child sits on his or her parent's lap, one green lamp is on the center wall, one red lamp is on the left side wall, and another red lamp is on the right side wall. Each trial begins by lighting the green lamp, which is then switched off when the child looks at it. Subsequently, one of the red lamps begins to blink. When the child turns his/her head to look at the blinking red light, the speech sound presentation begins. The sound continues until the child looks (turns) away from the light for more than 2 s, or until the presentation duration ends. The total looking times toward the target sounds and the other sounds are compared. If a significant difference is observed in looking time toward the two stimuli, the child is presumed to detect a difference between the two sounds.

A few past studies of infants aged <6 months used a non-nutritive high-amplitude sucking procedure (e.g., Floccia et al., 1997; Christophe and Morton, 1998; Nazzi et al., 1998). The typical sucking procedure is as follows. Initially, the infant's usual sucking rate (i.e., baseline rate) is checked. To hear the sound continuously, the infant must suck with a high-amplitude rate. When the infant begins to suck with a high rate, the target sound is presented. The stimulus sound is continuously presented until the sucking rate becomes lower than a predetermined threshold (e.g., 75 or 80% of the max rate of sucking) during the familiarization session or the test trial for 2 consecutive minutes. Usually, the sucking rate decreases with repeated or continuous presentation of the same stimulus, whereas the sucking rate increases immediately after presentation of a different stimulus. If a significant difference is observed in sucking rate toward the two sound stimuli, the infant is presumed to detect a difference between the two sounds.

Either method can indicate whether children detect differences between two sounds and show that infants and toddlers separate target sounds from the flow of speech, although these methods cannot indicate how infants and toddlers segment speech sounds. To understand clearly the mechanism by which infants and toddlers segment speech sounds, a new method is necessary.

The purpose of the present study was to establish a new method that can show directly how infants and toddlers segment speech sounds. Considering that syllable units are the fundamental components of language, this study assessed the utility of the new method by examining whether infants and toddlers can segment speech sounds based on syllable units. This new method used the revised intermodal matching procedure developed by Kobayashi et al. (2005). The outline of the present study was as follows.

In each trial of the familiarization session, the child was shown each ball in the display, accompanied by a speech sound. The ball dropped from the top to the bottom of the display. There were four trials in the familiarization session: one ball with one-syllable word sounds, two balls with two-syllable word sounds, three balls with three-syllable word sounds, and four balls with four-syllable word sounds.

In each trial of the test session, an opaque square appeared first in the middle of the display and extended to the bottom of the display. One-word sounds (one, two, three, or four syllables) were presented and the square receded toward the bottom of the display, then disappeared. The child immediately saw either the match condition, where the number of balls (e.g., three) matched the number of syllables presented aurally immediately prior (e.g., three-syllable words, te-re-bi), or the non-match condition, where the number of balls (e.g., two) did not match the number of syllables presented aurally immediately prior.

If the infants or toddlers correctly perceived the number of syllables concerning the relevant word sounds, we expected that the amount of time they spent looking toward the ball stimuli would differ between the match and non-match conditions. A significant difference in looking time during two conditions (matched familiar and non-matched novel conditions) has been regarded as evidence that a child can distinguish between them, and thus, that they can correctly perceive the number of stimuli (Gerken et al., 2015; DePaolis et al., 2016). The condition during which the child spends more time looking at the stimuli is not clear: some studies have reported that familiar stimuli received more attention (i.e., longer looking time) from infants and toddlers during modality-matching in a visual-auditory setting (e.g., Starkey et al., 1983; Golinkoff et al., 1987), whereas other studies have reported that unexpected events received greater attention from infants (Mix et al., 1997; Kobayashi et al., 2005). However, in a recent meta-analysis, Bergmann and Cristia (2016) examined the direction or condition in which children looked longer toward during the intermodal matching procedure. The data, which were collected with the intention of examining segmentation of word sounds, indicated that young children often spent longer looking at the stimuli in the familiar matched condition. Indeed, previous studies concerning attention toward stressed word sounds or one- or two-syllable word sounds found that infants and toddlers almost always looked longer toward the familiar sounds (e.g., Jusczyk et al., 1999b; Houston et al., 2000; Nishibayashi et al., 2015). Thus, in the present study, we presumed that the children would look longer toward the stimuli in the match condition than that in the non-match condition when they correctly perceived the number of syllables (in this case, basic morae).

We used this method to examine whether basic early segmentation based on syllable units or basic morae could be observed in infants and toddlers aged 6–25 months, regardless of linguistic skill level. One reason why we selected 6 months of age as the approximate earliest age to examine is because this is the point at which infants begin to detect differences in word sounds (Höhle et al., 2009; Bosch et al., 2013; Nishibayashi et al., 2015). The other reason is because this is the earliest age examined by studies of the bootstrapping hypothesis (e.g., Gleitman and Wanner, 1982; Jusczyk, 1997; Soderstrom et al., 2003; Bernard and Gervain, 2012; Wellmann et al., 2012). We selected 25 months as the approximate latest age at which children are expected to produce their first words for the following reason. Although the earliest children begin to speak is around 8 months, many children begin to speak after 1 year (Fenson et al., 1994). Word acquisition in typically developing Japanese children tends to be slightly delayed compared to English-learning children (Okumura et al., 2016), such that some typically developing Japanese children do not produce first words until around 24 months (Yoshioka and Tosa, 2014). Considering these data, it could be said that the age at which typically developing Japanese children acquire their first words ranges from 8 months to 2 years of age.

We assumed that, beginning at the age of ~6 months, children would segment word sounds in the same manner as older children. We did not expect age, linguistic ability, or sex to influence the segmentation of word sounds considering previous findings concerning the perception of prosodic features of speech sounds in very young children (e.g., Nazzi et al., 1998; Christophe et al., 2003; Bernard and Gervain, 2012; Wellmann et al., 2012). Thus, all participants' looking patterns toward the stimuli would be similar if the new method were to be useful for examining segmentation of speech sounds in those children.

## Methods

### Participants

Forty-five healthy monolingual Japanese infants or toddlers (21 boys and 24 girls; 6–25 months old; mean age = 13.6 months, standard deviation = 5.56 months) participated in this study, accompanied by their mothers. The data from five additional infants or toddlers were excluded from analysis because of fussiness during the task or < 30% looking time fixation during a test session (Tobii Pro Studio V3.2). All participants were recruited through flyers posted in various locations (e.g., nurseries and children's centers) and advertisements on the Internet homepage of our developmental laboratory. The participants and their parents lived in the Tokyo metropolitan area and had middle-class socioeconomic backgrounds. All participants, whose main caretakers were their mothers, were full-term born and monolingual. No child had been diagnosed with a developmental disability. All of the mothers were homemakers or on parental leave at the time of participation in this study.

Both verbal and written informed consent for the children to participate were provided by their mothers. In accordance with the Declaration of Helsinki, all procedures in the present study were approved by the Humanities and Social Sciences Research Ethics Committee of Ochanomizu University.

### Material

To check a child's language ability, the child's mother was asked to complete the Japanese version of the MacArthur Communicative Development Inventory regarding the child [the original version is known as the CDI (Fenson et al., 1993), while the Japanese version is known as the JCDI]. The inventory “Words and Gestures,” presumably suitable for assessing Japanese 8–18 month-old infants and toddlers (Ogura and Watamaki, 2004), was used for the present children aged 6–17 months (34 children including 15 boys and 19 girls; mean age = 10.8 months, standard deviation = 2.70 months). Although the inventory “Words and Gestures” was not developed for children as young as 6–7 months old, we used this inventory to confirm that the children in this age group had a lower linguistic level. Bergelson and Swingley (2012, 2015) used this inventory to check linguistic abilities in 6–9-month-old infants and 6–16-month-old infants in 2012 and 2015, respectively. The inventory “Words and Grammar,” presumably suitable for assessing Japanese 16–36-month-old infants and toddlers (Watamaki and Ogura, 2004), was used for the present children aged 18–25 months (11 children including six boys and five girls; mean age = 22.2 months, standard deviation = 2.18 months).

Werker et al. (2002) and Song et al. (2018) counted the raw number of words produced by toddlers (according to their mothers) using the MacArthur CDI (Fenson et al., 1993) and regarded this number as the toddler's vocabulary size. Bates and Goodman (1997) discussed vocabulary size in relation to the number of words the child understood and produced, according to the mother's description on the MacArthur CDI (Fenson et al., 1993). We thus determined each infant's or toddler's vocabulary size according to these approaches. The vocabulary size of children aged 6–17 months was determined by counting words that the child understood and produced, according to the mother's description on the JCDI inventory “Words and Gestures” (Ogura and Watamaki, 2004), whereas the vocabulary size of the children aged 18–25 months was determined by counting words the child produced, according to the mother's description on the JCDI inventory “Words and Grammar” (Watamaki and Ogura, 2004).

The word stimuli, which were the sounds presented to the participants, were chosen by referring to the Japanese version of the MacArthur Communicative Development Inventory (JCDI) and “Tables of vocabulary obtained from Japanese children by association method” (The National Research Institute Research Report 69, Tokyo-Shoseki Ltd. 1981). Acoustic measurements of pitch, pitch range, and duration for each of the word stimuli are shown in Table 1. Considering the characteristics of rhythm counting in young children (Takahashi, 1997, 2001), differences in CV structure and pitch within the usual range were not expected to influence rhythm counting for morae. The other details, such as the number of syllables (morae) within the word presented, are mentioned in the Procedure section.

**Table 1:** Average acoustic measurements for test stimuli.

**Stimuli**	**Mean pitch (Hz)**	**Mean pitch range (Hz)**	**Mean duration (s)**
Ki	254.48	38.36	0.34
me	240.65	33.37	0.32
a-si	222.65	66.49	0.61
ha-na	233.00	57.33	0.55
te-re-bi	215.72	106.7	0.70
mi-ru-ku	224.14	111.5	0.69
mi-so-si-ru	231.80	96.32	0.97
ku-tu-si-ta	243.18	127.3	0.90

### Apparatus and Stimuli

The infant or toddler was situated on their mother's lap in a chair. The stimuli were presented on a 17-inch laptop monitor (Dell Precision M6800), ~50–70 cm from the infant or toddler and 70 cm from the floor. The experimenter asked the mother for the child's eyes to be located at the level of the center of the monitor. This was checked by the experimenter immediately before the start of both familiarization and test sessions. The mother was also asked not to look at the display and not to interact with the child during the session. Presentation of the stimuli on the display were performed using Tobii Pro Studio V3.2. The child's fixation duration and gaze trajectory toward the stimuli or area of interest were recorded and measured using a Tobii X2-30 Compact Eye Tracker (display size 1920 × 1080 pixels) and Tobii Pro Studio V3.2. To confirm the child's visual attention and behavior for subsequent analyses, one video camera (Sony HDR-XR150) was also placed ~30–45 cm behind the monitor to record the child's visual attention and behavior.

Speech and chime sounds were presented through the laptop speaker. All children were confirmed to hear the sounds during the familiarization session. All speech sounds were produced by a single female native speaker who spoke standard Japanese and did not know the purpose of this study. The sounds were created as wav. files. All of the sound stimuli were presented at an average level of 65 dB.

### Procedure

The locations of experiments were determined by the children's mothers: either at the participants' homes or in our laboratory's playroom. Thus, the experiment was primarily conducted in our laboratory's playroom, but was conducted at the participants' homes in a few instances. In each situation, the child was relaxed and the surrounding environment was quiet. The child was accustomed to the playroom, the research setting, and the researcher through play interactions with the researcher before the experiment.

The revised version of the intermodal matching procedure developed by Kobayashi et al. (2005) was used in both familiarization and test sessions. In the first familiarization session of the experiment by Kobayashi et al. (2005), the infant saw two (or three) objects dropping from top to bottom in sequence, with a tone presented while each object dropped. In the second familiarization session of the experiment by Kobayashi et al. (2005), two (or three) auditory tones occurred in sequence during the period when two (or three) objects dropped from the top, although the movement of each object and the bottom remained hidden behind a screen. After the two (or three) objects had fully dropped, the screen was lowered and the two (or three) objects appeared. In this second familiarization session, the number of tones matched the number of objects that appeared after the screen was lowered. In the first portion of the test session in the experiment by Kobayashi et al. (2005), the infant heard two or three tones but the screen entirely covered the objects and their movement. In the second portion of the test session in the experiment by Kobayashi et al. (2005), the infant saw the screen lowered and two or three objects appeared. In that experiment, infants looked significantly longer at the unexpected event (e.g., two tones in the first portion of the test session and three objects in the second portion of the test session) than at the expected event (e.g., two tones in the first portion of the test session and two objects in the second portion of test session).

The present study used the revised versions of familiarization and test sessions from the experiment by Kobayashi et al. (2005) to examine whether children segmented speech sounds based on syllable units. The details of each session in the present study are explained below.

#### Familiarization Session

**Figure 1:**
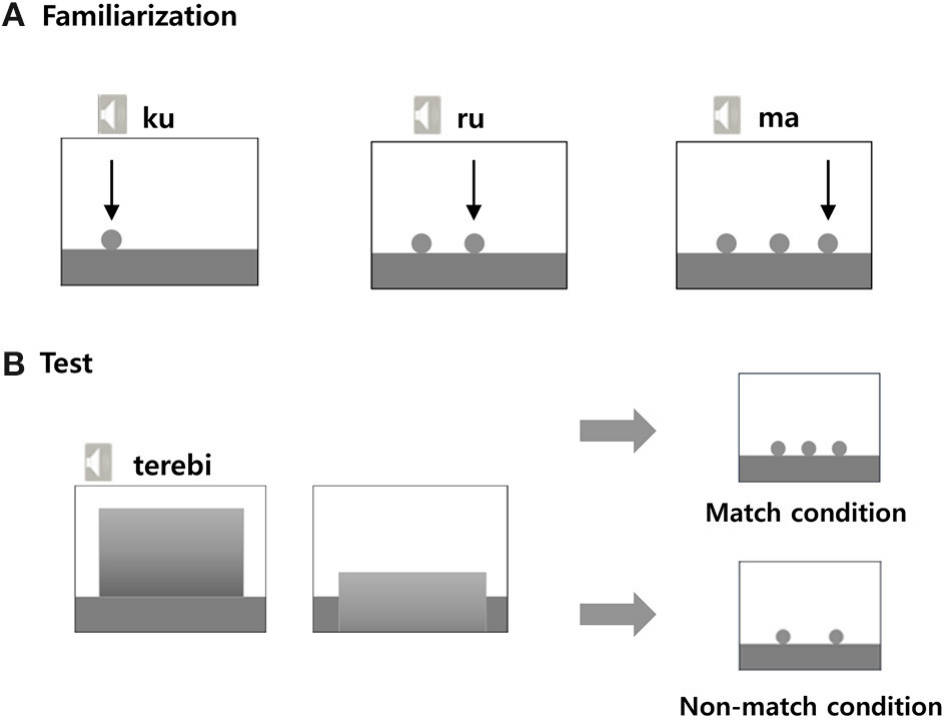
Presentation sequences in the three-syllable condition during the familiarization trial **(A)** and test trial **(B)**.

Five-point eye tracking calibration was initially conducted for each child, using Tobii Pro Studio V3.2. Following calibration, the familiarization session began. The familiarization session consisted of four trials. In each trial, the child was shown each sky blue ball dropping from the top to the bottom of the display (Figure 1A). Each ball was accompanied by a one-syllable speech sound while the ball dropped for 1 s. There were four types of words and trials. In the one-ball dropping trial, one ball dropped during presentation of a single one-syllable word sound. In the two-ball dropping trial, two balls dropped sequentially and each dropping ball was accompanied by a single one-syllable speech sound. Thus, two-syllable word sounds were presented with the two sequential dropping balls. In the three-ball dropping trial, three balls dropped sequentially and each dropping ball was accompanied by a single one-syllable speech sound at a rate of one-syllable sound per second. Thus, three-syllable word sounds were presented with the three sequential dropping balls, such that the first ball dropped with the sound [ku], the second ball dropped with the sound [ru], and the third ball dropped with the sound [ma] (Figure 1A). The four-ball dropping trial was conducted in a manner similar to that of the two-ball and three-ball dropping trials.

Details of the one-syllable, two-syllable, three-syllable, and four-syllable word sounds presented in each trial are shown in Table 2. The sounds were presented to each child in the order shown.

**Table 2:** Stimuli in familiarization session.

**Stimuli**	**Words and breaks**	**Meaning**	**Number of balls dropped**	**Presentation duration (s)**
1 syllable (1 mora)	e	Picture	1	5.5
2 syllables (2 morae)	u-mi	Sea	2	6.5
3 syllables (3 morae)	ku-ru-ma	Car	3	7.5
4 syllables (4 morae)	o-mu-re-tu	Omelet	4	8.5

#### Test Session

After the familiarization session, calibration was repeated and the test session began. The test session consisted of 16 trials: two one-syllable words × two conditions = four trials; two two-syllable words × two conditions = four trials; two three-syllable words × two conditions = four trials; and two four-syllable words × two conditions = four trials. There were three types of presentation orders during the 16 trials: A, B, and C (Table 3). Participants were randomized to each of the presentation orders (14 participants received order A, 15 participants received order B, and the remaining 16 participants received order C). Overall, each infant or toddler participated in 16 trials. The details of stimulus words and the numbers of presented balls in each ball presentation condition are shown in Table 4.

**Table 3:** Presentation orders.

**Order A**		**Order B**		**Order C**	
**Words and breaks**	**Condition**	**Words and breaks**	**Condition**	**Words and breaks**	**Condition**
mi-ru-ku	Match	ku-tu-si-ta	Match	ki	Non-match
ki	Non-match	ha-na	Non-match	mi-ru-ku	Match
a-si	Match	ki	Match	a-si	Match
te-re-bi	Non-match	mi-so-si-ru	Match	te-re-bi	Non-match
ku-tu-si-ta	Non-match	mi-ru-ku	Non-match	me	Match
ha-na	Match	te-re-bi	Match	ha-na	Non-match
a-si	Non-match	me	Non-match	te-re-bi	Match
me	Match	mi-so-si-ru	Non-match	mi-ru-ku	Non-match
ku-tu-si-ta	Match	mi-ru-ku	Match	mi-so-si-ru	Match
ha-na	Non-match	ki	Non-match	ku-tu-si-ta	Non-match
ki	Match	a-si	Match	mi-so-si-ru	Non-match
mi-so-si-ru	Match	te-re-bi	Non-match	me	Non-match
mi-ru-ku	Non-match	ku-tu-si-ta	Non-match	a-si	Non-match
te-re-bi	Match	ha-na	Match	ha-na	Match
me	Non-match	a-si	Non-match	ki	Match
mi-so-si-ru	Non-match	me	Match	ku-tu-si-ta	Match

**Table 4:** Stimuli and ball-presentation conditions in test session.

			**Number of balls in final presentation**	
**Stimuli**	**Words and breaks**	**Meaning**	**Match condition**	**Non-match condition**
1 syllable (1 mora)	ki	Tree	1	3
	me	Eye	1	4
2 syllables (2 morae)	a-si	Leg	2	3
	ha-na	Flower	2	4
3 syllables (3 morae)	te-re-bi	Television	3	2
	mi-ru-ku	Milk	3	1
4 syllables (4 morae)	mi-so-si-ru	Miso soup	4	2
	ku-tu-si-ta	Socks	4	1

In each trial, a yellowish-green square appeared first in the middle of the display and extended to the bottom of the display (Figure 1B). One-word sounds (one, two, three, or four syllables) were presented at natural speed for 2 s and the square receded toward the bottom of the display, then disappeared. Immediately, the infant or toddler saw either the match condition where the number of balls (e.g., three) matched the number of syllables presented aurally just prior (e.g., three-syllable words, te-re-bi) or the non-match condition where the number of balls (e.g., two) did not match the number of syllables presented aurally just prior. This final presentation of balls (in both match and non-match conditions) lasted 6 s (Figure 1B).

The infant's or toddler's looking times toward the stimuli in the final presentation of each trial during the test session were measured by Tobii Pro Studio V3.2 and used for the analyses. In detail, to record the amount of time the infants and toddlers spent looking at the balls on the floor in the lower part of the display, the experimenter set a rectangle within which the stimuli appeared (2500 mm width × 1450 mm height located right above the floor), using the built-in function in Tobii Pro Studio V3.2. The looking time included all traces recorded by the eye tracker that indicated that the children were looking inside the rectangle. If the children looked away from the rectangle, the gaze traces were not counted as part of the looking time.

As we explained in the Introduction section, considering meta-analyses of studies concerning young children's segmentation of word sounds (Bergmann and Cristia, 2016) and previous data concerning the detection of stressed word sounds and one- or two-syllable word sounds (e.g., Jusczyk et al., 1999a; Houston et al., 2000; Nishibayashi et al., 2015), we assumed that the children would spend more time looking toward the stimuli in the match condition than in the non-match condition if they correctly perceived the number of syllables (basic morae).

### Statistical Analysis

Most analyses were conducted using IBM SPSS Statistics, version 25. Cohen's d was calculated using Excel 2013. For the analyses, we used the ratio data, i.e., looking time toward the matched (or non-matched) stimuli/total looking time toward the stimuli (including looking time toward the matched and non-matched stimuli), to exclude the influence of differences in total looking time among the children and to ensure the homogeneity of variance between the variables to the greatest possible degree.

## Results

Although we did not expect to find any effects of sex, age, or linguistic ability, based on previous studies, we confirmed that these variables did not have significant effects.

We confirmed that there were no significant effects of sex in the ratio of looking time in the match condition [24 girls: mean = 0.54, standard deviation = 0.09; 21 boys: mean = 0.56, standard deviation = 0.14; t_(43)_ = 0.78, *p* = 0.44, d = 0.23, 95% confidence interval (−0.04, 0.10)]. Thus, we combined the data from the girls and boys for further analysis.

To check whether there was an effect of age, we calculated the correlation coefficient between age and the ratio of looking time toward the stimuli in the match condition. We found no significant correlation (*r* = −0.09, *p* = 0.56). Therefore, there appeared to be no effect of age on infants' and toddlers' segmenting of words based on syllable units.

Next, to check whether there was an effect of linguistic ability, we calculated the correlation coefficient between the linguistic score and the ratio of looking time toward the stimuli in the match condition. For the 34 children aged 6–17 months, the numbers of words understood and produced from the JCDI inventory “Words and Gestures” were used to determine a linguistic score. When we calculated the correlation coefficient between the linguistic score and the ratio of looking time toward the stimuli in the match condition, no significant correlation was observed (*r* = 0.13, *p* = 0.48). For the 11 children aged 18–25 months, the numbers of words understood and produced from the JCDI inventory “Words and Grammar” were used to determine a linguistic score. When we calculated the correlation coefficient between the linguistic score and the ratio of looking time toward the stimuli in the match condition, no significant correlation was observed (*r* = −0.003, *p* = 0.99). Thus, there appeared to be no effect of linguistic ability on infants' and toddlers' segmenting of words based on syllable units.

**Figure 2:**
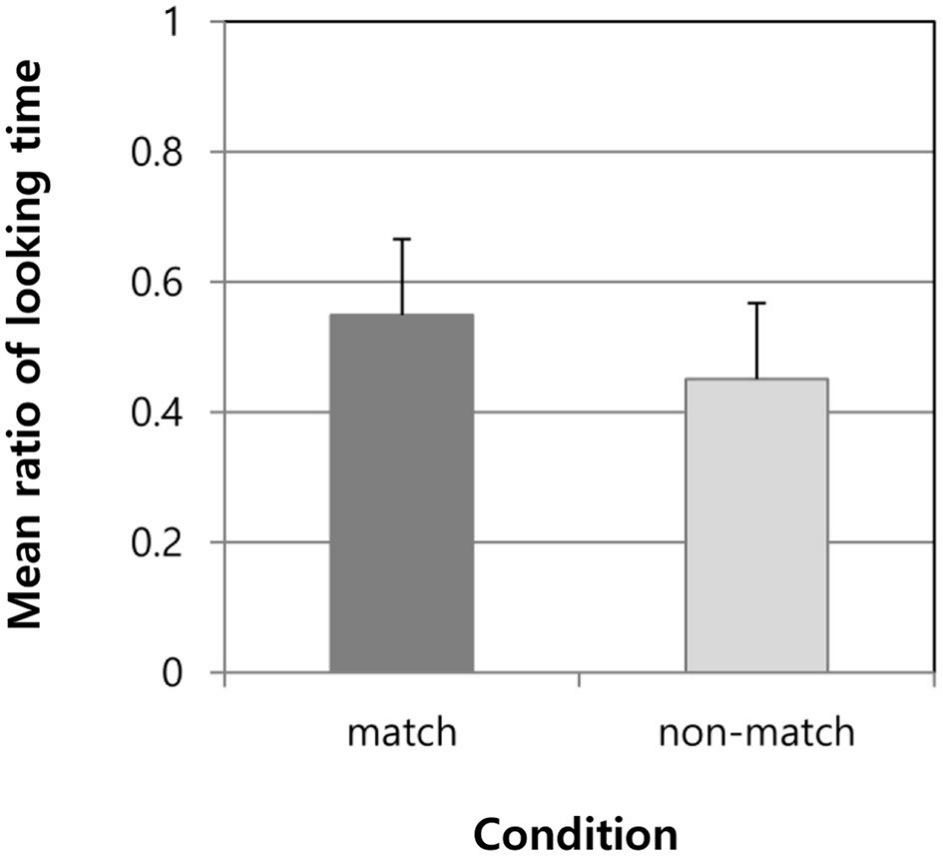
Mean ratio of looking times during test trials in matched and non-matched conditions.

We compared the ratio of looking time toward the ball stimuli between the match and non-match conditions during the final presentation in the test session. The children looked significantly longer at the stimuli in the match condition (mean = 0.55, standard deviation = 0.12) than those in the non-match condition (mean = 0.45, standard deviation = 0.12) [t_(44)_ = 2.75, *p* = 0.009, d = 0.82, 95% confidence interval (0.026, 0.166)] (Figure 2). This indicated that the children appropriately perceived the number of syllables (concurrent basic morae) included in Japanese words comprising basic morae.

Thus, these analyses implied that children aged 6 months to 2 years perceived word segmentation based on syllables or basic morae, regardless of sex, age, and linguistic ability.

## Discussion

The overall results of this study were as follows. Using a new intermodal matching procedure, we found that Japanese-learning infants and toddlers aged 6–25 months tended to segment Japanese words on the basis of concurrent basic mora units within syllable units because they looked significantly longer at visual stimuli in the matched condition than in the non-matched condition. No significant differences in age or linguistic ability were found in looking time toward the stimuli, thus indicating that the children segmented the words in a similar manner, regardless of age or linguistic ability. All the words comprised only basic morae, which are segmented in a manner similar to that of syllables. Thus, each word included the same numbers of basic morae and syllables. The results have three main implications.

First, the results suggest that this new intermodal matching procedure is more useful for determining whether and how young children segment words, compared with previous methods (e.g., the headturn preference procedure) because the new intermodal matching procedure can indicate the number of units that an infant or toddler perceives within a single word. Although the previous methods could indicate whether young children discriminate familiar monosyllabic or disyllabic words from novel monosyllabic or disyllabic words, they could not indicate the number of units perceived within the test words. This new method will facilitate expansion of knowledge concerning the segmentation of words by young children.

Second, the results indicate that the infants aged ≥6 months have begun to segment words using rhythmic cues in a manner similar to that of toddlers, suggesting that Japanese infants' and toddlers' use of syllable or mora units for word segmentation depends less on language abilities or experiences. The present results demonstrate this point more clearly, including the number of perceived units, compared with prior literature, thereby suggesting that infants and toddlers can detect sound differences based on rhythmic units. Additionally, the present results are consistent with a previous finding that 6-month-old infants could differentiate monosyllabic words and syllables embedded in disyllabic words based on syllable units (Nishibayashi et al., 2015).

Third, the results suggest that children tend to segment Japanese words, which include only basic morae, on the basis of concurrent basic mora units within syllable units. The syllable has been regarded as a salient unit of rhythmic structure for perception of speech sounds (Räsänen et al., 2018) and thus has been regarded as a universal unit during speech perception (Mehler, 1981; Dumay et al., 2002). The syllable structure is present in Japanese and many other languages (Greenberg, 1999). The results of the present study imply that the syllable serves as a basic unit for perception of speech sounds by Japanese-learning infants and toddlers. Accordingly, the use of syllable units for segmentation of speech sounds at an early age could be evident among many infants and toddlers learning various languages.

However, these results should be interpreted carefully. First, to gain a more comprehensive understanding of basic segmenting skills, further studies with larger samples and wider age ranges are necessary. Second, the present study used Japanese words that included only basic morae. As we mentioned in the Introduction section, although more than 70% of Japanese sound units are basic morae, which can be segmented in a manner identical to that of syllables, the other sound units, i.e., “special morae,” are segmented in a manner that differs from that of syllables. As we mentioned in the Introduction section, kitte (meaning stamp) includes three morae (ki, t, and te: “t” is a special mora unit, while “ki” and “te” are basic mora units) but two syllables: “t” is not counted as a syllable unit. Although Japanese has been included in a subcategory of syllable-timed rhythm languages (Otake et al., 1993; Arvaniti, 2009; Mazuka, 2009), different rhythms should be considered for special morae, compared with syllables. Perception and/or segmentation of special morae should be examined in Japanese-learning young children in future studies, especially as these young children begin to segment special morae based on unique mora units. Notably, Japanese-learning 4-year-old children may perceive special mora unit sound-like syllables because they often clap their hands based on syllable units (e.g., two claps for the word “kitte”), not on special mora units (e.g., three claps for the word “kitte”), whereas they vocalize words that include special morae sounds (Inagaki et al., 2000; Takahashi, 2001). Accordingly, Japanese-learning young children may segment special morae based on syllable units, rather than unique mora units, until 4 years of age. Infants and toddlers may therefore segment speech sounds based on syllable units, rather than rhythms specific to their mother tongue.

Moreover, it has not been fully investigated whether infants and toddlers whose native language belongs to the stress-timed category, such as English and German, segment speech sounds based on syllable-timed units. Previous methods only assessed whether infants and toddlers could detect differences in stress patterns between words. The new method described in this study will allow assessment of whether infants and toddlers learning a stress-timed language also segment speech sounds based on syllable-timed units. If those infants and toddlers are found to segment speech sounds based on syllable-timed units, that finding would imply that the segmentation of speech sounds based on syllable-timed units is a more innate tendency in young children.

Some consideration may be needed regarding the direction of preferential looking in the intermodal matching procedure. In the contexts of visual learning and visual testing, infants often prefer to look at novel stimuli (e.g., Fantz, 1961, 1964). However, the direction of preferential looking was not clearly established, especially in the contexts of visual learning and auditory (or tactile) testing. Familiar stimuli received greater attention (i.e., longer looking time) from infants in the usual modality-matching procedure (Starkey et al., 1983; Golinkoff et al., 1987), whereas unexpected novel events received greater attention from infants (Mix et al., 1997; Kobayashi et al., 2005). Although the direction of preferential looking toward stimuli is presumably influenced by experimental settings and conditions (Gerken et al., 2015; DePaolis et al., 2016), the biased direction toward stimuli observed using the new intermodal method also has important implications concerning infants' and toddlers' segmentation of speech sounds in future studies.

Finally, we would like to speculate regarding the relationship between the present segmenting skills and other prosodic skills and word acquisition. The segmentation of speech sounds is deeply linked to the perception of boundaries in sounds, which has led researchers to develop theories about sound units such as morphemes, strings, and phrases. As Soderstrom et al. (2003) suggested, the development of prosodic perception of morphemes, strings, and phrases may progress from more general perception of sounds to that of more language-specific sounds such that children acquire word sounds first and then develop knowledge about language. In the present study, we proposed a new method, which we found to be appropriate for examining the segmentation of speech sounds by children. This method may be useful in examining the relationships between skills regarding the segmenting and structuring of sound-units. During the course of development, perceptual skills regarding speech sounds likely move from more general to more language-specific as a child acquires words and develops knowledge about language. However, this process is ambiguous, and so further studies are needed to clarify these mechanisms.

To the best of our knowledge, this study is the first to demonstrate empirically that the new intermodal matching procedure is useful for examining segmentation of speech sounds by young children, and that Japanese-learning children aged 6–25 months segmented Japanese words (comprising basic mora sounds) on the basis of concurrent basic mora units within syllable units, regardless of their ages and linguistic abilities. The findings will lead to further developments concerning infants' and toddlers' speech sound perception and language development.

## Data Availability Statement

Datasets generated for this study are available on request to the corresponding authors.

## Ethics Statement

The procedures of the present study were approved by the Humanities and Social Sciences Research Ethics Committee of Ochanomizu University (Ethics approval number: 2017-63). Both verbal and written informed consent for the children to participate were provided by the participants' legal guardian/next of kin.

## Author Contributions

YC and IU: substantial contributions to the concept and design of the study and to the acquisition, analysis, and interpretation of data. YC and IU: drafting of the manuscript and revising it critically for important intellectual content. Both authors contributed to manuscript revision and have read and approved the submitted version.

## Conflict of Interest

The authors declare that the research was conducted in the absence of any commercial or financial relationships that could be construed as a potential conflict of interest.
